# Supplementary material for: The meiotic LINC complex component KASH5 is an activating adaptor for cytoplasmic dynein
Source: J Cell Biol. 2023 Mar 22;222(5):e202204042. doi: 10.1083/jcb.202204042 (PMC10071310; doi:10.1083/jcb.202204042)
Supplement: Table S1 — shows quantitation of immunoblots shown in Fig 1 C and Fig. 3 E. [file JCB_202204042_TableS1.docx]

**Table S1. Quantitation of immunoblots shown in Figs 1C and 3E**

|  | Fig. 1C. GFP-KASH5DK or GFP-nesprin2aDK pull-downs | | Fig. 3E. GFP-KASH5DK or GFP-nesprin2aDK pull-downs in siRNA-treated cells | | |
| --- | --- | --- | --- | --- | --- |
| Pull-down condition | GFP-KASH5DK | GFP-N2aDK | GFP-KASH5DK control kd | GFP-KASH5DK LIC1&2 kd | GFP-N2aDK control kd |
| IC | 103.6 | 0.1 | 100 | 3.4 | 0.2 |
| LIC1 | 74.4 | 0.4 | 100 | 1.1 | 0.1 |
| LIC2 | 78.3 | 0.6 | 100 | 0.04 | 0.1 |
| p150 | 20.4 | 0.2 | 100 | 2.9 | 2.3 |
| LIS1 | 37.1 | 1.3 |  |  |  |
| BICD2 | 0.5 | 0.0 |  |  |  |

For Fig. 1C, the values were calculated as (bound-prey/input-prey)/(bound-GFP/input-GFP)*100. Since the Fig. 3E data were from siRNA-treated cells the bound/input ratio of prey protein was not used. Instead, the amount of protein present in the GFP-KASH5DK pull-down in control siRNA-treated cells was set at 100%, and the prey protein present in the other pull-downs were expressed relative to that. In Fig. 3E, the knock-down efficacy for LIC1 and LIC2 was 85%.
