# Supplementary material for: The meiotic LINC complex component KASH5 is an activating adaptor for cytoplasmic dynein
Source: J Cell Biol. 2023 Mar 22;222(5):e202204042. doi: 10.1083/jcb.202204042 (PMC10071310; doi:10.1083/jcb.202204042)
Supplement: Table S2 — shows multinomial logistic regression statistical analysis of Golgi apparatus morphology following LIC depletions and rescue by GFP and GFP-LIC1 constructs. [file JCB_202204042_TableS2.docx]

**Table S2. Multinomial logistic regression statistical analysis of Golgi apparatus morphology following LIC depletions and rescue by GFP and GFP-LIC1 constructs.**

The data presented graphically in Figure 5C and E were analysed by multinomial logistic regression, which compares the most likely control phenotype with those observed in test samples. The odds of obtaining each phenotype, relative to the commonest normal phenotype (cluster/ribbon), was calculated for each experimental condition compared to the odds in the reference condition of either control siRNA treatment (for Figure 5C), or LIC1 and 2 double depletion followed by rescue with full length GFP-LIC1 (for Figure 5E). Analysis of 100 cells per condition, in each of 3 independent experiments. The change in odds, 95% confidence intervals and P value that the change is significant are shown in each case. Grey fill indicates non-significant comparisons.

| **Figure 5 C analysis** | **Cluster/ribbon** | **Broken ribbon** | **Semi-scatter** | **Full scatter** | **Key** |
| --- | --- | --- | --- | --- | --- |
| Control siRNA vs. LIC1 kd | (1.0)  n/a  n/a | 37.4  22-64  <0.0001 | 5.4  1.7-17  0.004 | 3.3  0.7-17  0.144 | Change in odds  95% CI  P value |
| Control siRNA vs. LIC2 kd | (1.0)  n/a  n/a | 20.0  11.9-33.7  <0.0001 | 10.4  3.9-28  <0.0001 | 2.3  0.4-11.8  0.301 | Change in odds  95% CI  P value |
| Control siRNA vs. LIC 1 and 2 kd | (1.0)  n/a  n/a | 33.6  8-140  <0.0001 | 601  137-2631  <0.0001 | 8104  1617-40617  <0.0001 | Change in odds  95% CI  P value |
| **Figure 5 E analysis** |  |  |  |  |  |
| control siRNA vs. LIC kd + GFP rescue | (1.0)  n/a  n/a | 8.0  2.7-17  <0.00001 | 34  15-76  <0.00001 | 230  99-534  <0.00001 | Change in odds  95% CI  P value |
| control siRNA vs. LIC kd + LIC1 FL rescue | (1.0)  n/a  n/a | 1.28  0.9-1.8  0.179 | 0.72  0.4-1.3  0.276 | 0.86  0.4-1.8  0.690 | Change in odds  95% CI  P value |
| control siRNA vs. LIC kd + LIC1 CT2 rescue | (1.0)  n/a  n/a | 1.05  0.7-1.5  0.800 | 1.12  0.7-1.9  0.665 | 1.24  0.6-2.5  0.540 | Change in odds  95% CI  P value |
| control siRNA vs. LIC kd + LIC1 CT3 rescue | (1.0)  n/a  n/a | 4.9  2.8-8.7  <0.00001 | 10.5  5.5-10.1  <0.00001 | 90  46-179  <0.00001 | Change in odds  95% CI  P value |
| LIC kd with LIC1 FL rescue vs. GFP rescue | (1.0)  n/a  n/a | 6.3  2.9-13  <0.00001 | 47  21-109  <0.00001 | 268  112-640  <0.00001 | Change in odds  95% CI  P value |
| LIC kd with LIC1 FL rescue vs. LIC1 CT2 rescue | (1.0)  n/a  n/a | 0.82  0.6-1.2  0.282 | 1.6  0.9-2.8  0.135 | 1.4  0.7-3.0  0.320 | Change in odds  95% CI  P value |
| LIC kd with LIC1 FL rescue vs. LIC1 CT3 rescue | (1.0)  n/a  n/a | 3.9  2.2-6.8  <0.00001 | 14.5  7.3-29  <0.00001 | 105  51-215  <0.00001 | Change in odds  95% CI  P value |
| LIC kd with LIC1 CT2 rescue vs. control siRNA | (1.0)  n/a  n/a | 0.78  0.5-1.1  0.179 | 1.4  0.8-2.5  0.276 | 1.2  0.6-2.4  0.690 | Change in odds  95% CI  P value |
