# Supplementary material for: The meiotic LINC complex component KASH5 is an activating adaptor for cytoplasmic dynein
Source: J Cell Biol. 2023 Mar 22;222(5):e202204042. doi: 10.1083/jcb.202204042 (PMC10071310; doi:10.1083/jcb.202204042)
Supplement: Table S3 — shows multinomial logistic regression statistical analysis of Golgi apparatus morphology following expression of GFP-KASH5ΔK wild-type and EF-hand mutants. [file JCB_202204042_TableS3.docx]

**Table S3. Multinomial logistic regression statistical analysis of Golgi apparatus morphology following expression of GFP-KASH5ΔK wild-type and EF-hand mutants.**

The data presented graphically in Figure 8E were analysed by multinomial logistic regression, which compares the most likely control phenotype with those observed in test samples. The odds of obtaining each phenotype, relative to the commonest normal phenotype, was calculated for each experimental condition compared to the odds in the reference condition. The reference conditions were the commonest normal Golgi apparatus phenotype (cluster/ribbon), in either GFP or GFP-KASH5ΔK-AA expressing cells. Analysis of 100 cells per condition, in each of 3 independent experiments. The change in odds, 95% confidence intervals and P value that the change is significant are shown in each case. Grey fill indicates non-significant comparisons.

| **Condition** | **Cluster/ribbon** | **Broken ribbon** | **Semi-scatter** | **Full scatter** | **Key** |
| --- | --- | --- | --- | --- | --- |
| GFP vs.  GFP-KASH5ΔK-WT | (1.0)  n/a  n/a | 17.2  4.4-66.5  <0.0001 | 362  95-1388  <0.0001 | 3796  684-21073  <0.0001 | Odds ratio  95% CI for odds  P value |
| GFP vs.  GFP-KASH5ΔK-*fue* | (1.0)  n/a  n/a | 5.6  3.8-8.3  <0.0001 | 6.1  3.5-10.5  <0.0001 | 7.4  1.9-29  0.004 | Odds ratio  95% CI for odds  P value |
| GFP vs.  GFP-KASH5ΔK-AA | (1.0)  n/a  n/a | 5.1  3.4-7.5  <0.0001 | 7.7  4.5-13.2  <0.0001 | 7.4  1.8-29.2  0.004 | Odds ratio  95% CI for odds  P value |
| GFP vs.  GFP-KASH5ΔK-Mod 1 | (1.0)  n/a  n/a | 4.8  3.3-7.1  <0.0001 | 6.1  3.6-10.6  <0.0001 | 4.6  1.1-19.9  0.040 | Odds ratio  95% CI for odds  P value |
| GFP vs.  GFP-KASH5ΔK-Mod 2 | (1.0)  n/a  n/a | 6.6  1.8-24.3  0.004 | 201  59.2-683  <0.0001 | 4142  823-20859  <0.0001 | Odds ratio  95% CI for odds  P value |
| GFP-KASH5ΔK-AA vs. GFP-KASH5ΔK-*fue* | (1.0)  n/a  n/a | 1.1  0.7-1.7  0.643 | 0.8  0.5-1.3  0.366 | 1.0  0.3-3.0  0.995 | Odds ratio  95% CI for odds  P value |
| GFP-KASH5ΔK-AA vs. GFP-KASH5ΔK-Mod 1 | (1.0)  n/a  n/a | 1.3  0.4-4.8  0.694 | 26.1  7.8-87  <0.0001 | 562  142-2232  <0.0001 | Odds ratio  95% CI for odds  P value |
| GFP-KASH5ΔK-AA vs. GFP-KASH5ΔK-Mod 2 | (1.0)  n/a  n/a | 1.0  0.6-1.4  0.832 | 0.8  0.5-1.3  0.381 | 0.6  0.2-2.1  0.443 | Odds ratio  95% CI for odds  P value |
| GFP-KASH5ΔK-AA vs. GFP | (1.0)  n/a  n/a | 0.2  0.1-0.3  <0.0001 | 0.13  0.08-0.2  <0.0001 | 0.14  0.03-0.54  0.004 | Odds ratio  95% CI for odds  P value |
| GFP-KASH5ΔK-AA vs. GFP-KASH5ΔK-WT | (1.0)  n/a  n/a | 3.4  0.9-13.2  0.081 | 47  12-177  <0.0001 | 515  116-2291  <0.0001 | Odds ratio  95% CI for odds  P value |
