# Supplementary material for: The meiotic LINC complex component KASH5 is an activating adaptor for cytoplasmic dynein
Source: J Cell Biol. 2023 Mar 22;222(5):e202204042. doi: 10.1083/jcb.202204042 (PMC10071310; doi:10.1083/jcb.202204042)
Supplement: Table S4 — shows multinomial logistic regression statistical analysis of changes in early endosome and lysosome distribution after LIC depletion and rescue by GFP and GFP-LIC1 constructs. [file JCB_202204042_TableS4.docx]

**Table S4. Multinomial logistic regression statistical analysis of changes in early endosome and lysosome distribution after LIC depletion and rescue by GFP and GFP-LIC1 constructs.**

The data presented graphically in Figure S3 C and D were analysed by multinomial logistic regression, which compares the most likely control phenotype with those observed in test samples. The odds of obtaining each phenotype, relative to the commonest normal phenotype, was calculated for each experimental condition compared to the odds in the reference condition. The reference conditions were the commonest normal phenotype (clustered) in either control siRNA treatment, or LIC1 and 2 double depletions followed by rescue with full length GFP-LIC1. Analysis of 100 cells per condition, in each of 3 independent experiments. The change in odds, 95% confidence intervals and P value that the change is significant are shown in each case. Grey fill indicates non-significant comparisons.

| **Figures S3C** | **Central endosome cluster** | **Scattered endosomes** | **Peripheral endosomes** | **Peripheral endosome cluster** | **Key** |
| --- | --- | --- | --- | --- | --- |
| Control siRNA vs. LIC kd with GFP rescue | (1.0)  n/a  n/a | 64.8  20-210  <0.0001 | 4851  950-14759  <0.0001 | 1410  269-7394  <0.0001 | Change in odds  95% CI  P value |
| Control siRNA vs. LIC kd with FL LIC1 rescue | (1.0)  n/a  n/a | 0.56  0.4-0.8  0.001 | 0.82  0.2-4.1  0.812 | 0.82  0.16-4.1  0.809 | Change in odds  95% CI  P value |
| Control siRNA vs. LIC kd with LIC1 CT2 rescue | (1.0)  n/a  n/a | 1.47  1.0-2.0  0.001 | 6.45  1.8-22.8  0.004 | 1.7  0.4-7.9  0.478 | Change in odds  95% CI  P value |
| Control siRNA vs. LIC kd with LIC1 CT3 rescue | (1.0)  n/a  n/a | 64.5  28-150  <0.0001 | 956  230-3970  <0.0001 | 195  43-874  <0.0001 | Change in odds  95% CI  P value |
| LIC kd with FL LIC1 rescue vs. LIC1 CT2 rescue | (1.0)  n/a  n/a | 2.6  18-3.7  <0.0001 | 7.8  2.2-28  0.001 | 2.1  0.46-9.7  0.333 | Change in odds  95% CI  P value |
| LIC kd with FL LIC1 rescue vs. LIC1 CT3 rescue | (1.0)  n/a  n/a | 115  49-270  <0.0001 | 1163  280-4829  <0.0001 | 238  53-1068  <0.0001 | Change in odds  95% CI  P value |
| LIC kd with FL LIC1 rescue vs. GFP rescue | (1.0)  n/a  n/a | 115  35-376  <0.0001 | 5899  1155-30129  <0.0001 | 1721  328-9036  <0.0001 | Change in odds  95% CI  P value |
| LIC kd with FL LIC1 rescue vs. control siRNA | (1.0)  n/a  n/a | 1.8  1.2-2.5  0.001 | 1.2  0.24-6.1  0.812 | 1.2  0.24-6.2  0.809 | Change in odds  95% CI  P value |
| **Figure S3E** | **Central lysosome cluster** | **Scattered lysosomes** | **Peripheral lysosomes** | **Peripheral lysosome cluster** | **Key** |
| Control siRNA vs. LIC kd with GFP rescue | (1.0)  n/a  n/a | 38  21-68  <0.0001 | 331  92-1190  <0.0001 | 32  7.3-144  <0.0001 | Change in odds  95% CI  P value |
| Control siRNA vs. LIC kd with FL LIC1 rescue | (1.0)  n/a  n/a | 0.77  0.5-1.1  0.149 | 2.04  0.5-8.3  0.318 | 1.0  0.2-5.1  0.985 | Change in odds  95% CI  P value |
| Control siRNA vs. LIC kd with LIC1 CT2 rescue | (1.0)  n/a  n/a | 0.97  0.69-1.4  0.870 | 1.8  0.4-7.6  0.427 | 1.1  0.2-5.4  0.931 | Change in odds  95% CI  P value |
| Control siRNA vs. LIC kd with LIC1 CT3 rescue | (1.0)  n/a  n/a | 15.5  9.9-14  <0.0001 | 165  49-555  <0.0001 | 14.7  3.5-62  <0.0001 | Change in odds  95% CI  P value |
| LIC kd with FL LIC1 rescue vs. LIC1 CT2 rescue | (1.0)  n/a  n/a | 1.3  0.9-1.8  0.209 | 0.9  0.27-2.9  0.835 | 1.1  0.2-5.3  0.947 | Change in odds  95% CI  P value |
| LIC kd with FL LIC1 rescue vs. LIC1 CT3 rescue | (1.0)  n/a  n/a | 20.2  12.7-32  <0.0001 | 81  32-202  <0.0001 | 14.5  3.4-61  <0.0001 | Change in odds  95% CI  P value |
| LIC kd with FL LIC1 rescue vs. GFP rescue | (1.0)  n/a  n/a | 49  27-90  <0.0001 | 162  60-440  <0.0001 | 32  7.2-141  <0.0001 | Change in odds  95% CI  P value |
| LIC kd with FL LIC1 rescue vs. control siRNA | (1.0)  n/a  n/a | 1.3  0.9-1.9  0.149 | 0.5  0.1-2.0  0.318 | 1.0  0.2-4.9  0.985 | Change in odds  95% CI  P value |
