# Supplementary figures and images for: The meiotic LINC complex component KASH5 is an activating adaptor for cytoplasmic dynein
Source: J Cell Biol. 2023 Mar 22;222(5):e202204042. doi: 10.1083/jcb.202204042 (PMC10071310; doi:10.1083/jcb.202204042)

## Source data for Garner et al. Figure 1

**C**

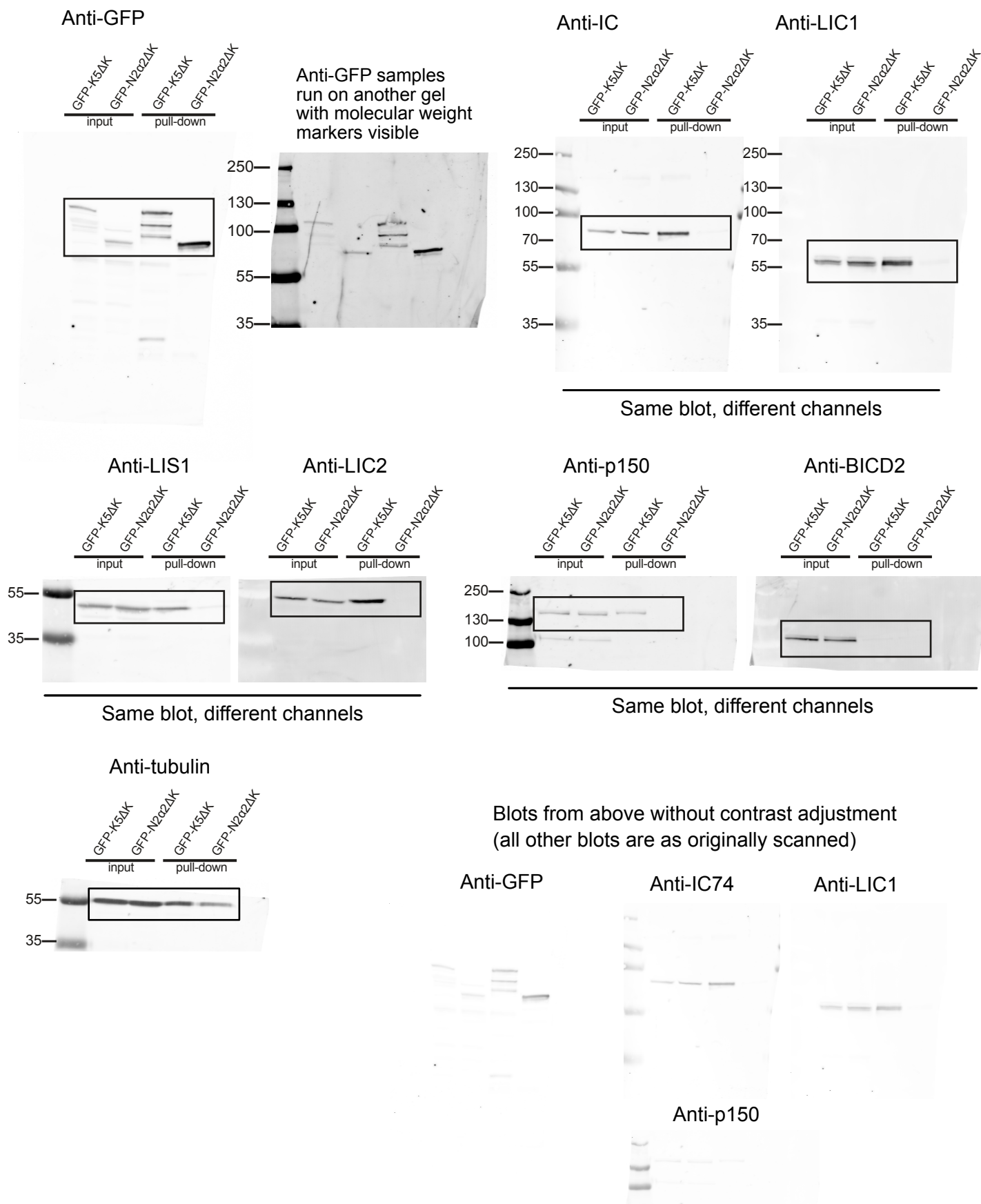

## Source data for Garner et al. Figure 1

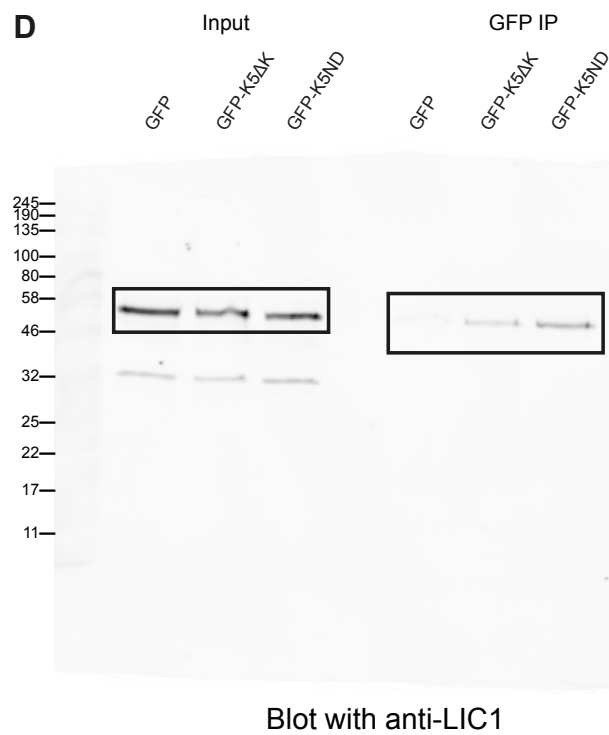

Supplement: SourceData F1 — is the source file for Fig. 1. [file JCB_202204042_SourceDataF1.pdf]

Garner et al. Source data for figure 3E

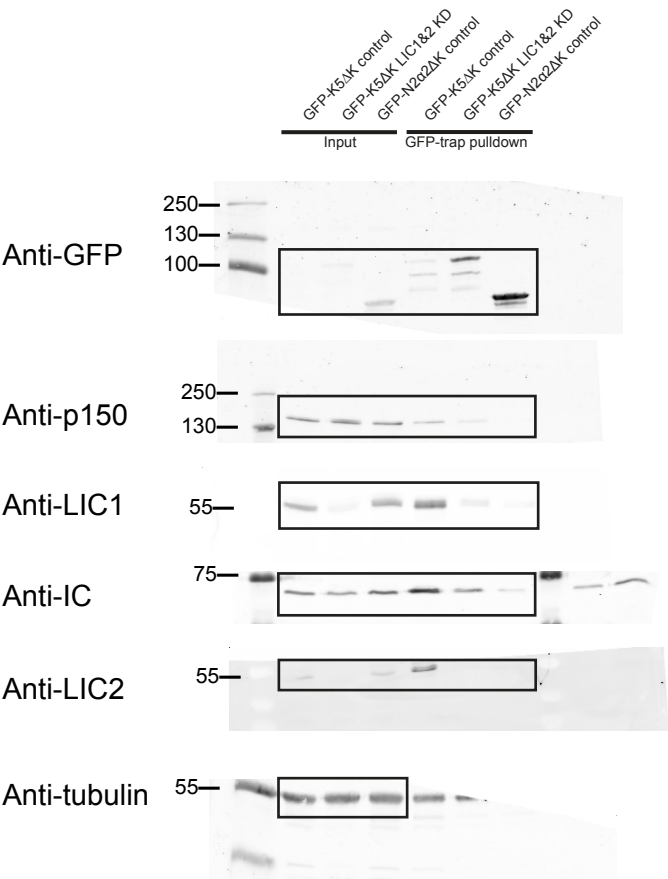

Supplement: SourceData F3 — is the source file for Fig. 3. [file JCB_202204042_SourceDataF3.pdf]

Source data for Garner et al. Figure 4

**B**

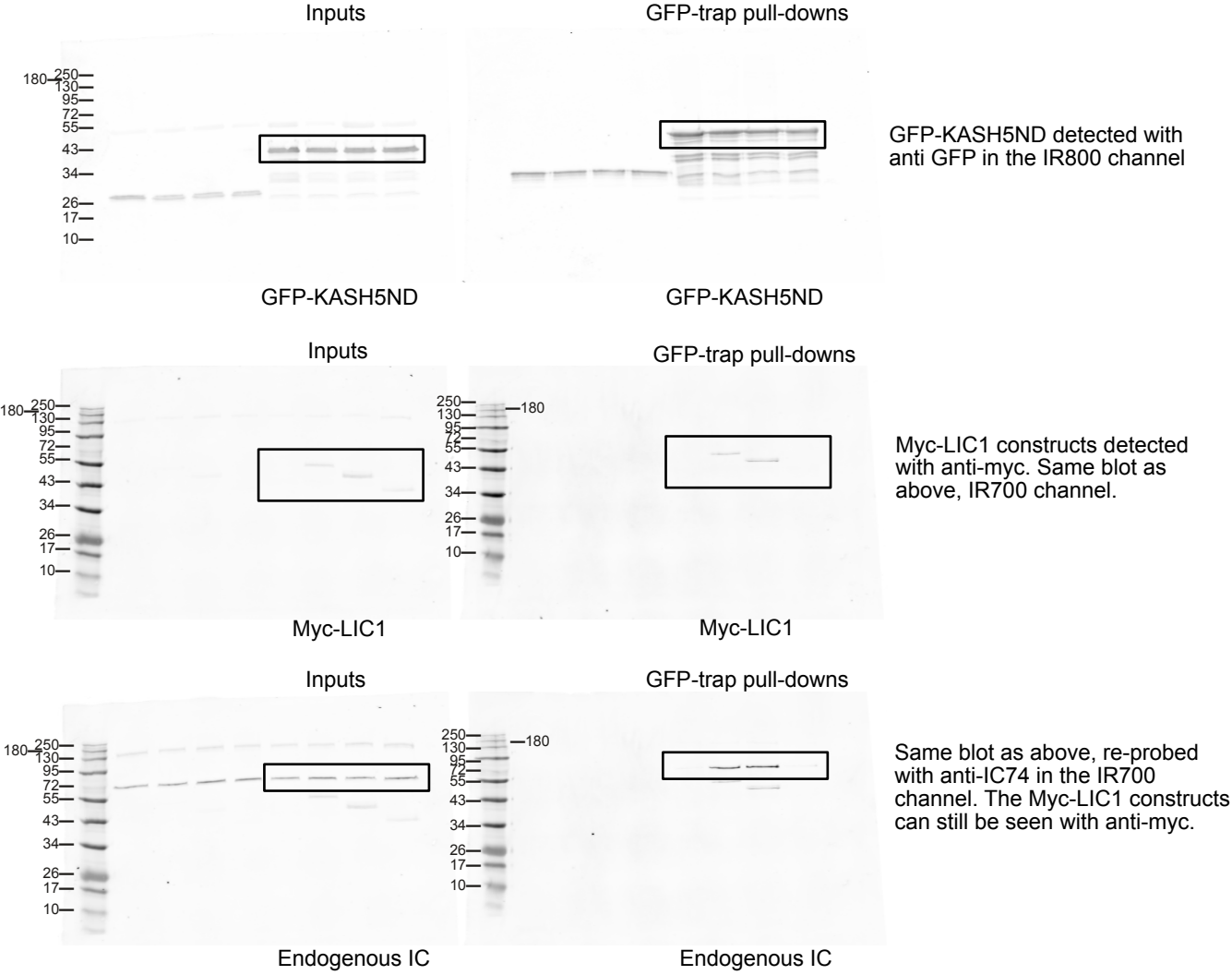

**C**

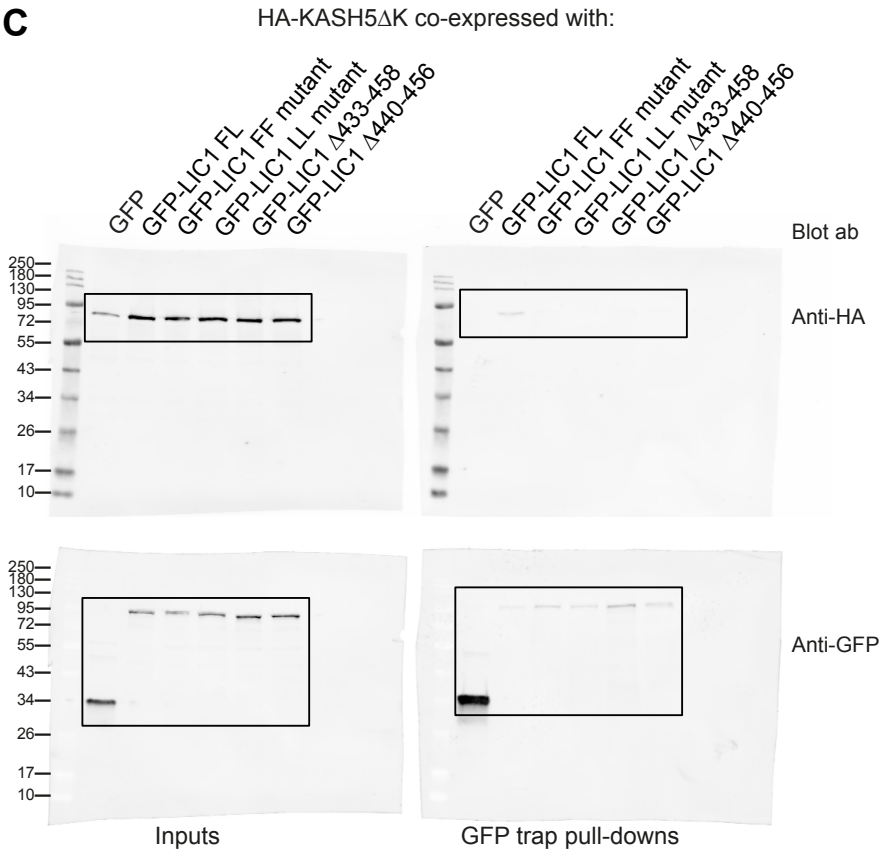

Supplement: SourceData F4 — is the source file for Fig. 4. [file JCB_202204042_SourceDataF4.pdf]

## Source data for Figure 5, Garner et al.

**A**

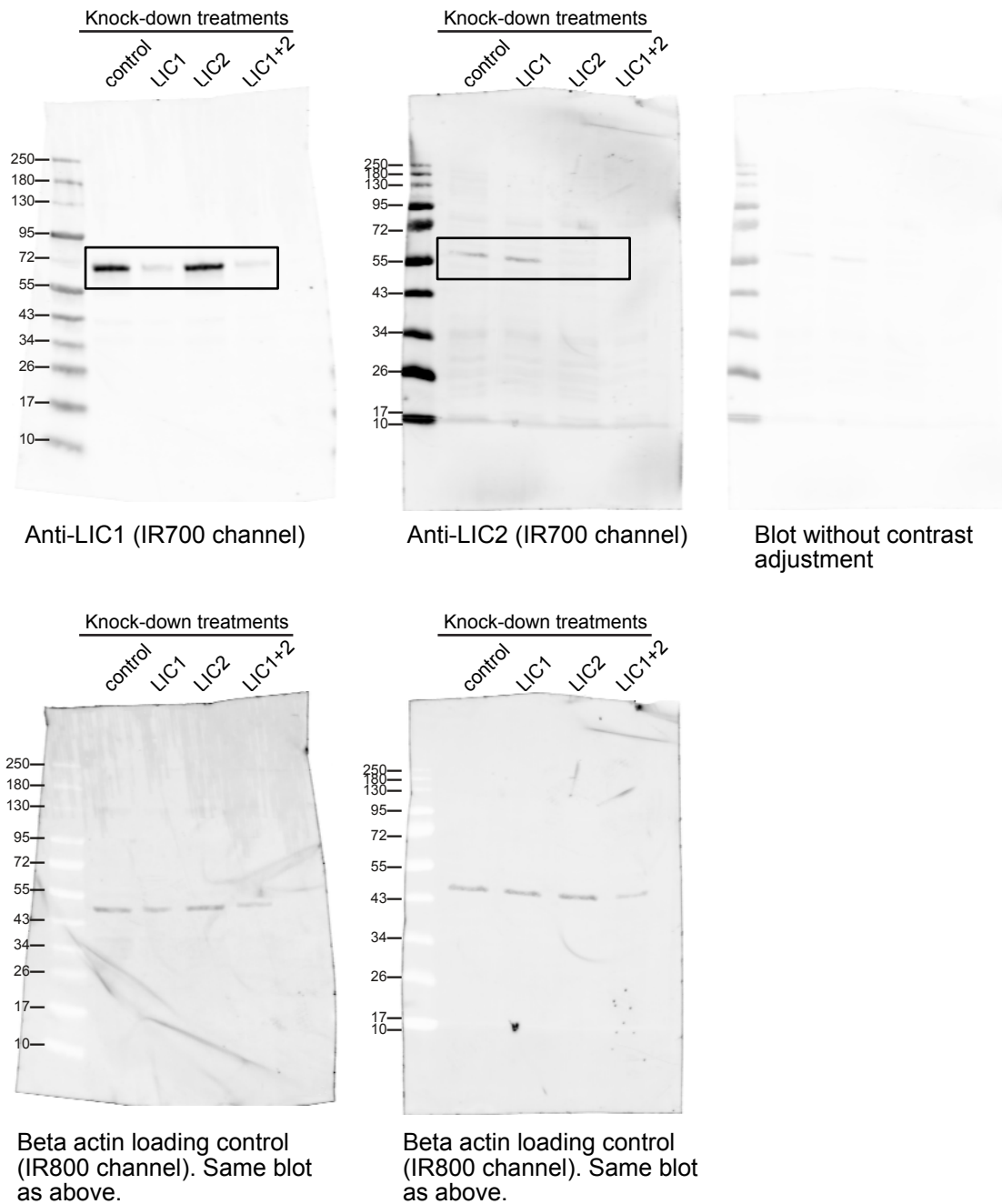

Supplement: SourceData F5 — is the source file for Fig. 5. [file JCB_202204042_SourceDataF5.pdf]

Source data for Figure 7A, Garner et al.

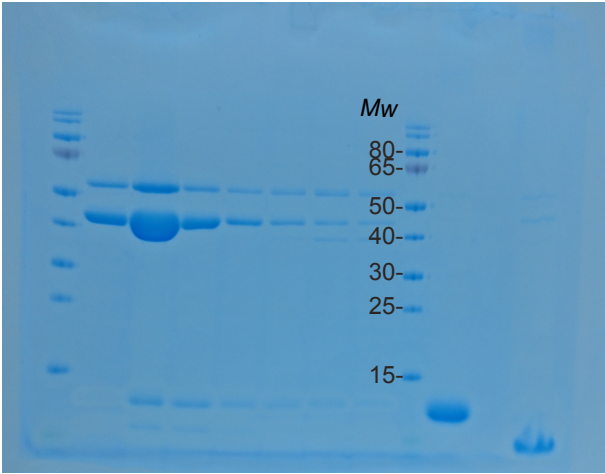

KASH5  
1-115

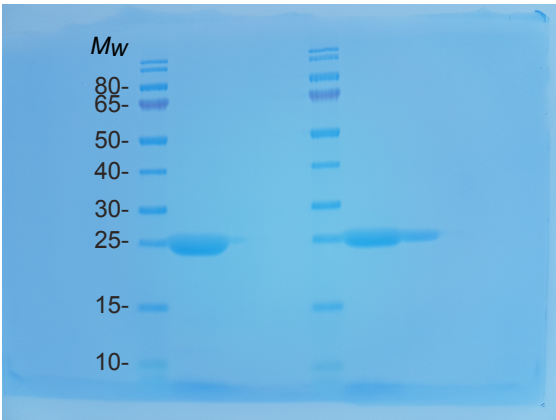

KASH5  
155-349

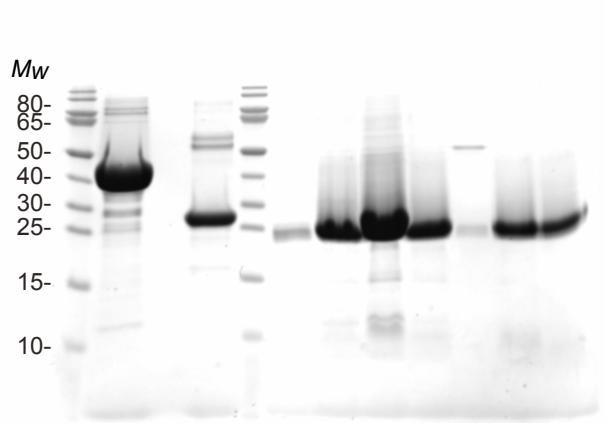

KASH5  
1-349

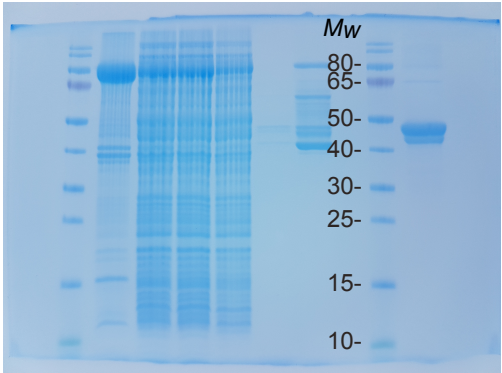

KASH5  
1-407

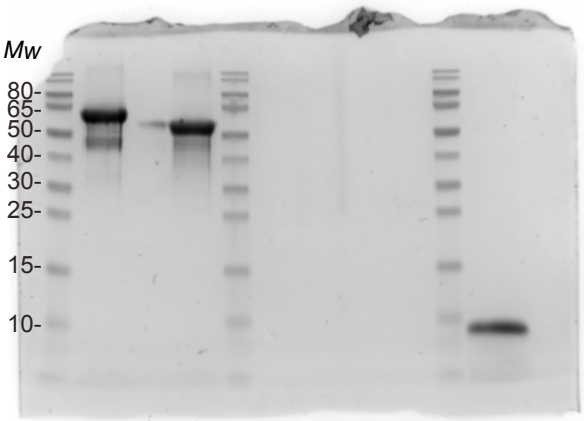

KASH5 KASH5  
1-507 1-460

Supplement: SourceData F7 — is the source file for Fig. 7. [file JCB_202204042_SourceDataF7.pdf]

Source data for Garner et al. Figure 8

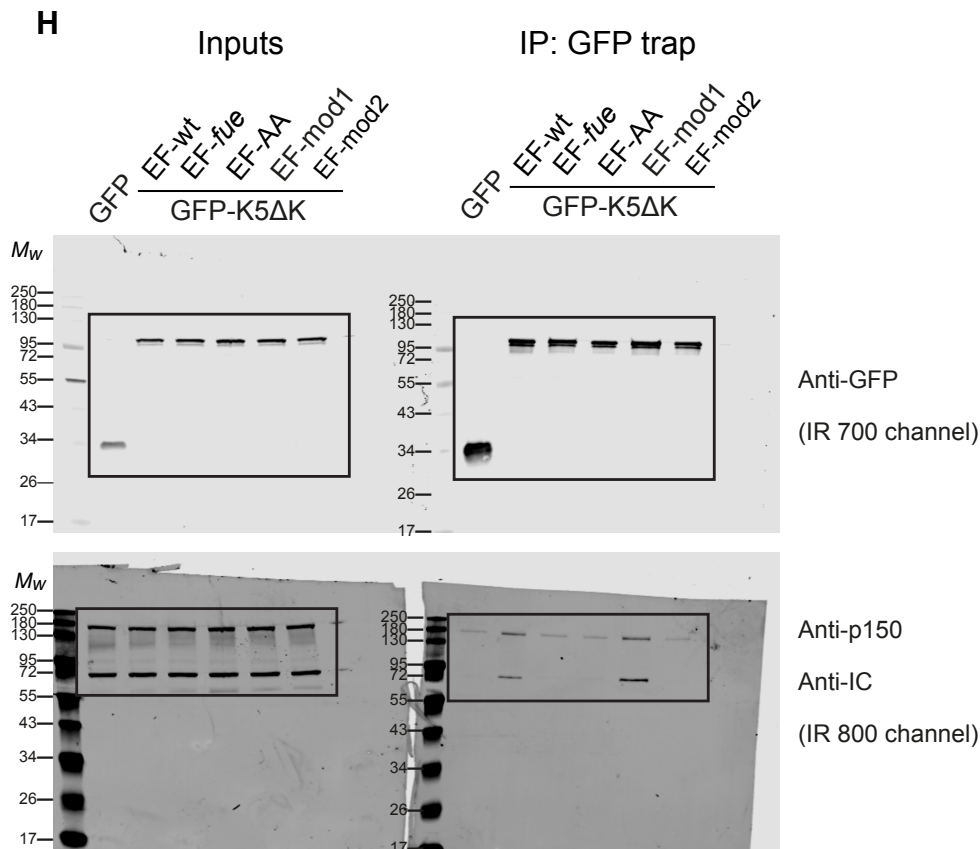

Blots without contrast adjustment

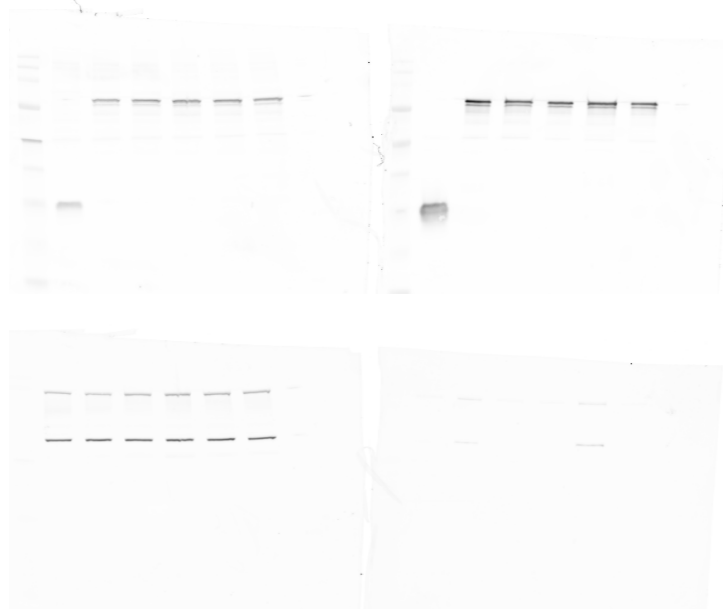

Supplement: SourceData F8 — is the source file for Fig. 8. [file JCB_202204042_SourceDataF8.pdf]

Source data for Garner et al. Figure S2

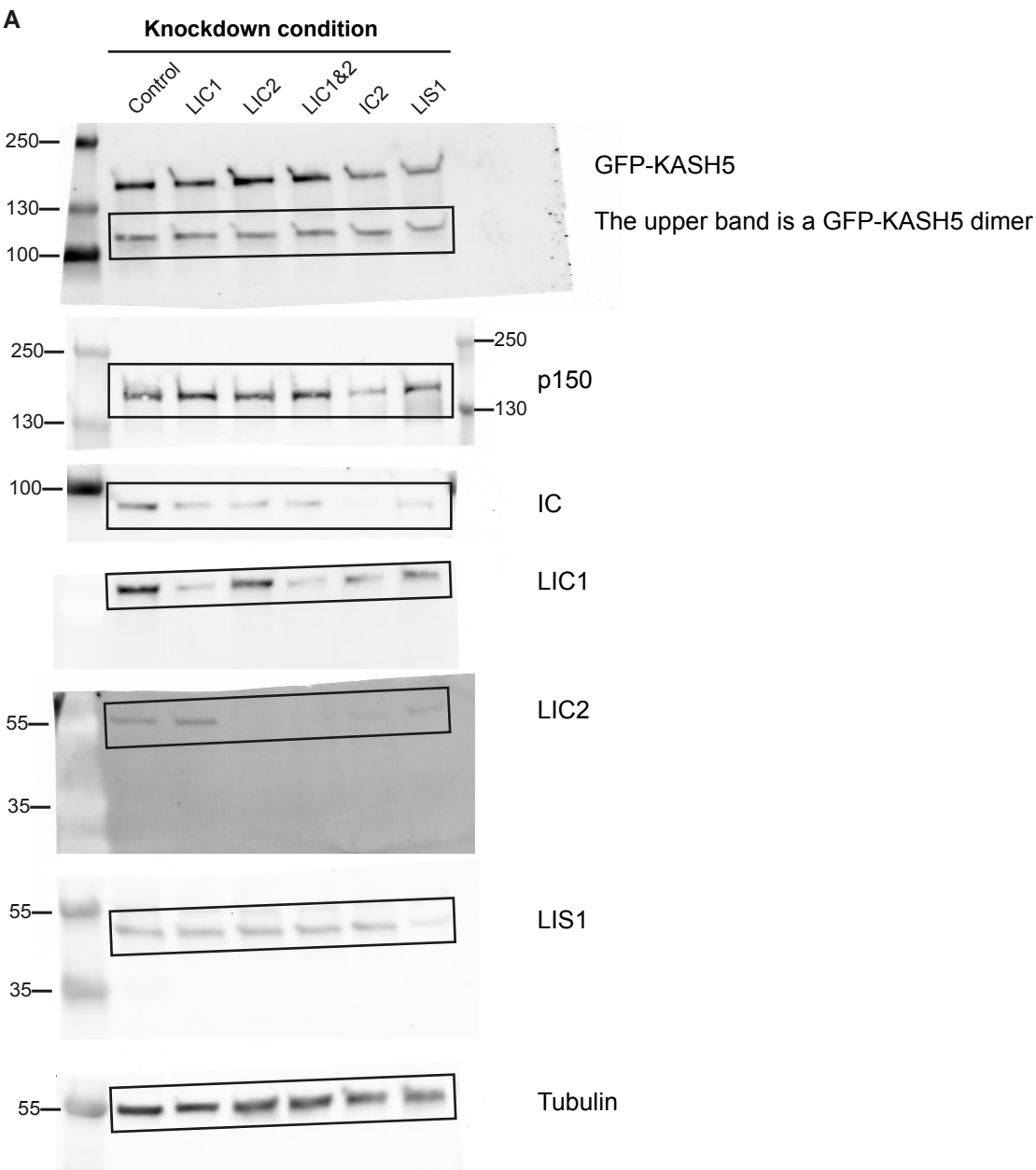

Source data for Garner et al. Figure S2

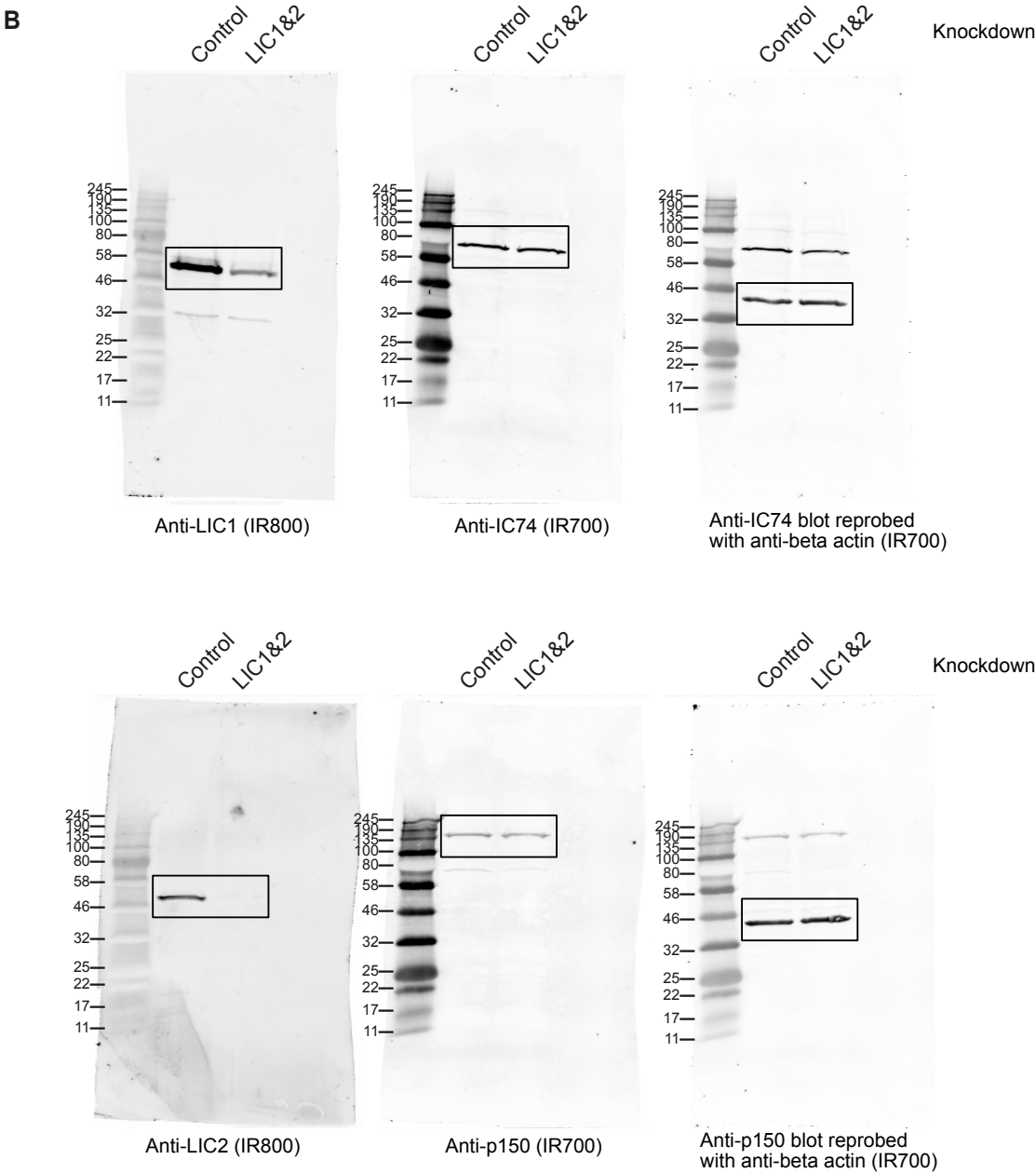

Supplement: SourceData FS2 — is the source file for Fig. S2. [file JCB_202204042_SourceDataFS2.pdf]
